# 

**Published:** 1862-04

**Authors:** 


### BOOKS RECEIVED.

*Anatomy, Descriptive and Surgical, by* HENRY GRAY, F. R. S., *Fellow of the Royal College of Surgeons, and Lecturer on Anatomy at St. George's Hospital Medical School. The drawings by* H. V. CARTER, M. D., *late Demonstrator of Anatomy at St. George's Hospital. The dissectings, jointly by the author and* DR. CARTER. *Second American from the revised and enlarged London edition, with three hundred and ninety-five engravings on wood. Philadelphia:* BLANCHARD & LEA.

---

*A Book about Doctors by* J. CORDY JEFFERSON. *Reprinted from the English edition. New York:* RUDD & CARLTON, 130 *Grand Street.— London*: HURST & BLACKETT.

### PAMPHLETS RECEIVED.

*Introductory Address delivered at the opening of the course on Physiology, in the Medical Department of the University of the Pacific, at San Francisco, Cal., by* DR. L. C. LANE, *Prof. of Physiology.*

*Report of Sanitary Commission on the subject of amputations through the foot and at the ankle-joint.*

*Transactions of the New York Academy of Medicine.*

*A Sermon preached at the funeral of* Elson Carr, M. D., *by the pastor,* Rev. O. E. Daggett.

*Harpers' New Monthly Magazine, for April. Published by* Harper Bro's., *Franklin Square, New York.*

*Peterson's Ladies' National Magazine, for April. Published by* Chas. J Petrrson, 306 *Chestnut Street, Philadelphia.*

